# Targeting accelerated pulmonary ageing to treat chronic obstructive pulmonary disease‐induced neuropathological comorbidities

**DOI:** 10.1111/bph.16263

**Published:** 2023-11-15

**Authors:** Simone N. De Luca, Ross Vlahos

**Affiliations:** ^1^ Centre for Respiratory Science and Health, School of Health & Biomedical Sciences RMIT University Melbourne Victoria Australia

**Keywords:** accelerated ageing, cellular senescence, chronic obstructive pulmonary disease, circadian rhythm, neurodegeneration

## Abstract

Chronic obstructive pulmonary disease (COPD) is a major incurable health burden, ranking as the third leading cause of death worldwide, mainly driven by cigarette smoking. COPD is characterised by persistent airway inflammation, lung function decline and premature ageing with the presence of pulmonary senescent cells. This review proposes that cellular senescence, a state of stable cell cycle arrest linked to ageing, induced by inflammation and oxidative stress in COPD, extends beyond the lungs and affects the systemic circulation. This pulmonary senescent profile will reach other organs via extracellular vesicles contributing to brain inflammation and damage, and increasing the risk of neurological comorbidities, such as stroke, cerebral small vessel disease and Alzheimer's disease. The review explores the role of cellular senescence in COPD‐associated brain conditions and investigates the relationship between cellular senescence and circadian rhythm in COPD. Additionally, it discusses potential therapies, including senomorphic and senolytic treatments, as novel strategies to halt or improve the progression of COPD.

**Abbreviations**
8‐OHdG: 8‐hydroxydeoxyguanosine
8‐OHG: 8‐hydroxyguanosine
BBB: blood–brain barrier
BMAL1: brain and muscle ARNT‐like protein‐1
CLOCK: circadian locomotor output cycle kaput
COPD: chronic obstructive pulmonary disease
DDR: DNA damage response
IPF: idiopathic pulmonary fibrosis
JAK: Janus‐activated kinases
MMP: matrix metalloproteinase
PPE: porcine pancreatic elastase
SASP: senescence‐associated secretory phenotype
SA‐β‐gal: senescence‐associated beta‐galactosidase
SVD: small vessel disease
VCID/VaD: vascular cognitive impairment and dementia

## 1 INTRODUCTION

Chronic obstructive pulmonary disease (COPD) poses a significant global health challenge, currently ranking as the third leading cause of death worldwide (WHO, 2023). COPD is characterised by persistent respiratory symptoms and irreversible airflow limitation (Alvar et al., 2023). The global burden of COPD amounted to 251 million cases, resulting in a staggering mortality rate of up to 3.23 million deaths in 2019 (WHO, 2023). Importantly, more than 90% of COPD‐related deaths occur in low‐ and middle‐income countries due to higher levels of cigarette smoke and non‐smoking household air pollution such as biomass fuel used for cooking and heating (Salvi & Barnes, 2010). In industrialised nations, the majority of COPD cases stem from long‐term exposure to cigarette smoke, while occupational dusts, chemicals, traffic‐related air pollution and airborne irritants also contribute to the development of the disease (Alvar et al., 2023).

COPD‐induced airway obstruction is commonly associated with increased inflammation in airway tissues and in bronchoalveolar lavage fluid, as shown by significant increases in macrophages and neutrophils (Di Stefano et al., 1998; Pesci et al., 1998), elevated pro‐inflammatory cytokines (such as TNF‐α, IL‐1β) and chemokines (CCL2, CCL3) (Barnes & Celli, 2009), cell apoptosis (Churg et al., 2008), oxidative stress (reactive oxygen species [ROS] and reactive nitrogen species) and suppression of antioxidant proteins (such as glutathione peroxidase, catalase and superoxide dismutase) (Bernardo et al., 2015) in both the airways and lung parenchyma, in response to noxious particles and gases (Churg et al., 2008). This chronic low‐grade inflammation results in destruction of the alveolar wall and hypersecretion of mucus, thus causing structural damage to both small and large airways (bronchitis) and lung parenchyma (emphysema) (Barnes & Celli, 2009) subsequently provoking a decline in lung function (Bhatt et al., 2016). In addition, mediators of the inflammation and oxidative stress in the lung may enter the systemic circulation resulting in significant increases in cytokines, chemokines, oxidative stress, and acute phase proteins, including C‐reactive protein and fibrinogen, in individuals with COPD, compared to healthy equivalents (Leuzzi et al., 2017). Such systemic inflammation is particularly evident when the disease is severe and during acute bacterial and/or viral exacerbations of COPD (Barnes & Celli, 2009). Notably, there is compelling evidence that inflammation may become established in distal tissue which in turn may initiate or worsen other chronic medical conditions, commonly referred to as comorbid disorders, such as skeletal muscle wasting (Chan et al., 2020), cardiovascular disease (Sussan et al., 2009), metabolic‐syndrome and bone disease (Chen et al., 2006) and cognitive impairments (Borson et al., 2008; De Luca et al., 2022). These COPD comorbidities may potentiate the morbidity of COPD, leading to increased hospitalisations, mortality and healthcare costs.

## 2 ASSOCIATION OF COPD WITH NEUROPATHOLOGICAL DISORDERS

Cigarette smoking has been correlated with the pathogenesis and/or development of numerous major neurological disorders including stroke, and small vessel disease (SVD) (Kim et al., 2018; Portegies et al., 2016; Söderholm et al., 2016). Moreover, individuals with COPD are predisposed to neurodegenerative diseases, including but not limited to, mild cognitive impairment, dementias such as Alzheimer's disease and vascular cognitive impairment and dementia (VCID/VaD), multiple sclerosis and mental health disorders (Dodd et al., 2010; Lutsey et al., 2019; Tondo et al., 2018; Villeneuve et al., 2012). Despite the limited literature, it could be hypothesised that the pro‐inflammatory and oxidative stress profiles within the COPD lungs reaches the systemic circulation, triggering a loss of blood–brain barrier (BBB) function and integrity, leading to an over‐exuberant inflammatory profile within the CNS, thus augmenting the pathogenesis and progression of these neuropathological diseases.

### 2.1 Clinical studies of neurological disorders in COPD

Extensive research has demonstrated a higher prevalence of neurological disorders, including stroke and SVD, among individuals with COPD, when compared to the general population. Notably, COPD patients exhibit a significantly heightened incidence of all‐cause stroke, with a 20%–30% increased risk, compared to their healthy counterparts (Kim et al., 2018; Portegies et al., 2016; Söderholm et al., 2016). Furthermore, within the first two years following a COPD diagnosis, the risk escalates by as much as 46% (Söderholm et al., 2016). Specifically, the nationwide study from Sweden found that individuals with COPD had a higher risk of ischemic (20%), haemorrhagic (29%) and subarachnoid haemorrhage (46%) strokes compared to healthy age‐equivalents (Söderholm et al., 2016). Similarly, after adjusting for age and smoking status, the Rotterdam Study demonstrated that individuals with COPD have a higher risk of both ischemic (13%) and haemorrhagic (53%) strokes (Portegies et al., 2016). A comprehensive meta‐analysis encompassing eight longitudinal studies revealed that individuals diagnosed with COPD had an increased stroke risk in comparison to healthy controls, with a significant increase of 30% (Kim et al., 2018). Moreover, the occurrence of bacterial and/or viral exacerbations in COPD patients raises the risk to an alarming extent, reaching up to 7 times that of control subjects (Portegies et al., 2016). Hospitalisation resulting from these exacerbations has been correlated with increased overall mortality rates for acute myocardial infarction and ischemic stroke (Wang et al., 2020).

COPD has emerged as a significant contributor to SVD, often manifested through the presence of cerebral microbleeds and haemosiderin deposits in macrophages, indicative of partly undigested ferritin and lysosomes. Notably, individuals with COPD exhibit a substantial 14% increase in cerebral microbleeds compared to healthy controls. Additionally, a longitudinal study found that 10.9% of individuals with COPD, who were initially free of cerebral microbleeds, developed microbleeds within 3.4 years, signifying a higher risk compared to the general population, which carries a 2.6% risk (Lahousse et al., 2013).

### 2.2 Clinical studies of neurodegenerative diseases in COPD

The intricate relationship between COPD and its effects on the cerebrovasculature has been correlated with an array of neurodegenerative disorders. These encompass, among others, mild cognitive impairment, Alzheimer's disease, VCID/VaD and Parkinson's disease, highlighting the broad impact of COPD beyond its respiratory manifestations. It is now well understood that people with COPD suffer from cognitive dysfunction, with up to 61% experiencing cognitive losses compared to only 12% of the age‐equivalent healthy population (Dodd et al., 2010; Villeneuve et al., 2012). These people often suffer from impairments in memory, executive function and attention. The duration of stable COPD symptoms and pulmonary pathologies have been correlated with the prevalence of cognitive impairment (Singh et al., 2014; Yazar et al., 2018), with people suffering from more severe COPD symptoms, compared to mild‐to‐moderate disease, showing worse cognitive function (Li et al., 2013; Yin et al., 2019). People with exacerbations of COPD have significantly worsened cognitive function, when compared with stable COPD and healthy participants (Crisan et al., 2014; France et al., 2021).

In addition to the increased risk for cognitive impairments in COPD, this population also have an increased risk for developing neurodegenerative diseases including Alzheimer's disease, and Parkinson's disease. An observational study by Laio et al. demonstrated that people with COPD are 1.74 (74%) times more likely to develop dementia (such as Alzheimer's disease or Parkinson's disease) compared with age‐ and gender‐matched controls (Liao et al., 2015). Patients with COPD and Alzheimer's disease have worse executive functioning, compared to patients with only Alzheimer's disease (Tondo et al., 2018). The large retrospective observational Atherosclerosis Risk in Communities Study reported an odds ratio of 1.24 (24%) for Alzheimer's disease in people with COPD compared with controls (Lutsey et al., 2019). Interestingly, a recent study examining the causal relationship between COPD and Alzheimer's disease, found that neither reduced lung function nor COPD phenotype was causally associated with an increased risk of Alzheimer's disease (Higbee et al., 2021) indicating that this association may be due to shared risk factors.

## 3 ACCELERATED AGEING IN COPD

As we age, our cells undergo a process termed cellular senescence, which is considered a hallmark of ageing. Cellular senescence involves the permanent withdrawal of cells from the cell cycle, leading to the cessation of cellular division, while the cells remain metabolically active, but unresponsive to mitogenic stimuli (Gorgoulis et al., 2019). Cells can undergo senescence due to various cellular responses to internal and external stress, resulting in different types of senescence, such as replicative senescence, oncogene‐induced senescence, stress‐induced senescence or mitochondrial DNA damage‐induced senescence (Gorgoulis et al., 2019). There are common signalling pathways and hallmarks associated with cellular senescence. These include disturbances in autophagy and lysosomal function, DNA damage, impairment of proteostasis (maintenance of protein homeostasis), reduced propensity for apoptosis (programmed cell death), and increased secretion of pro‐inflammatory cytokines and their mediators. This increased secretion of pro‐inflammatory molecules is commonly referred to as the senescence‐associated secretory phenotype (SASP) (Gorgoulis et al., 2019). While acute cellular senescence is deemed beneficial during embryonic development, protection against tumour malignancy and wound healing/tissue repair (Gorgoulis et al., 2019), the accumulation of senescent cells during ageing can lead to tissue dysfunction, age‐related diseases, and shortened lifespan. Transplanting senescent cells into elderly mice resulted in increased organ failure and mortality (Xu et al., 2018), whereas eliminating naturally occurring senescent cells increased the lifespan of normally ageing mice (Baker et al., 2016;).

**FIGURE 1:**
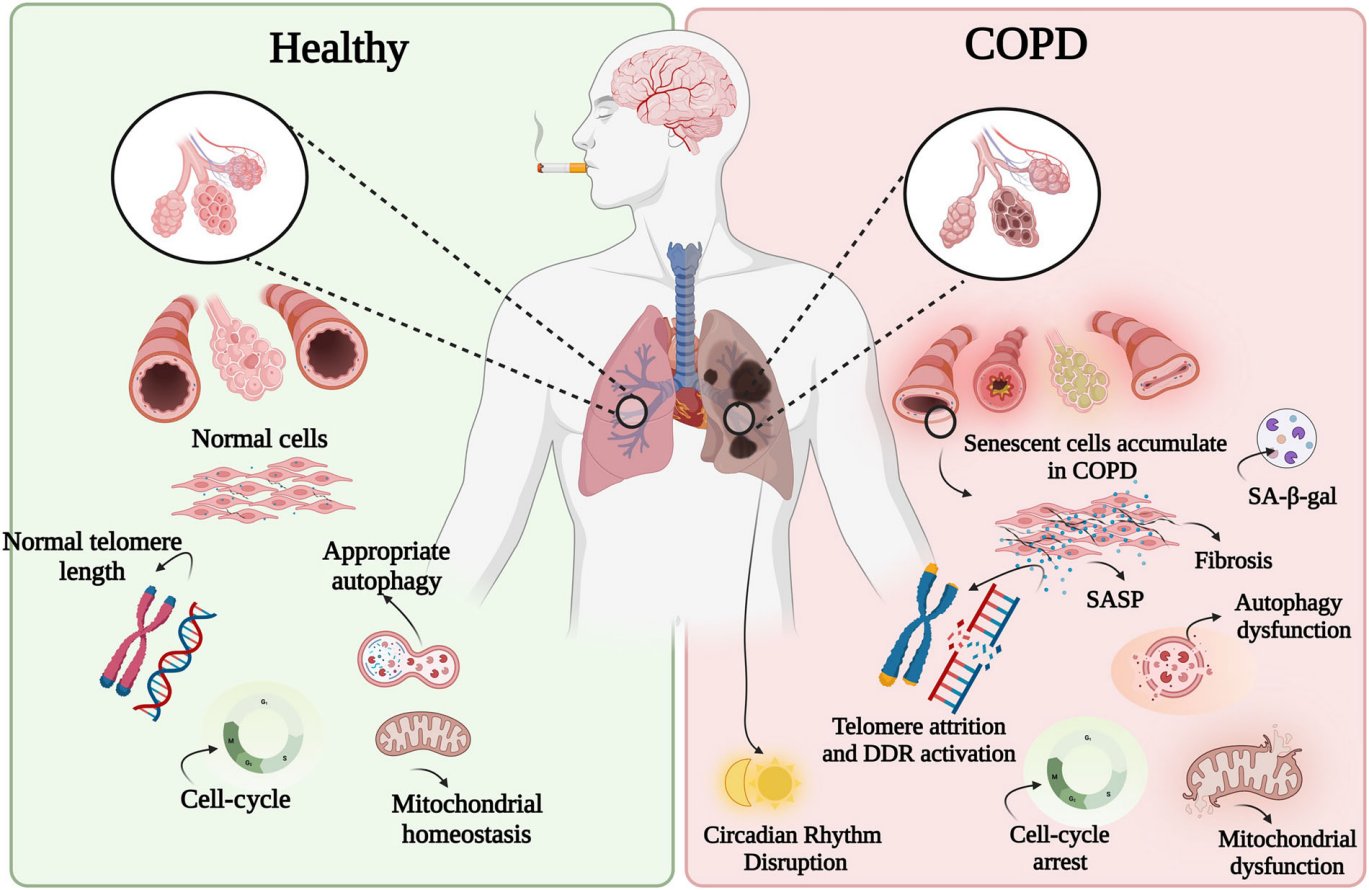
Proposed mechanism of accelerated ageing in chronic obstructive pulmonary disease (COPD) induced by exposure to cigarette smoke. On the left, features of a normal lung during healthy ageing are shown, a low number of senescent cells with homeostatic cell cycle, typical mitochondrial and autophagy function, and standard telomere lengths. On the right, altered features in lungs of cigarette smoke‐induced COPD are shown, associated with fibrosis, increased senescent cells and key characteristics of ageing including telomere attrition, DNA damage response (DDR) activation, elevated secretion rates of senescence‐associated secretory phenotype (SASP), cell‐cycle arrest, mitochondrial dysfunction, autophagy dysfunction and circadian rhythm disruption. We hypothesise that SASP mediators (such as inflammatory cytokines, chemokines and growth factors) from senescent pulmonary cells will be exported by cells via extracellular vesicles into the systemic circulation causing damage to the brain leading to the development of neuronal and immune cell accelerated ageing and neuropathological comorbidities.

Premature or accelerated ageing has been linked to many pulmonary diseases, including COPD and idiopathic pulmonary fibrosis (IPF). Thus, an increased understanding of molecular pathways involved in ageing and how this process is accelerated in COPD is crucial for identifying new therapeutic targets. The hallmarks of ageing can be categorised into two areas: those affecting cellular processes (such as cellular senescence, stem cells exhaustion and altered intercellular and intracellular communication), and those affecting metabolism (including mitochondrial dysfunction, loss of proteostasis, and dysregulated nutrient sensing) (López‐Otín et al., 2013; Meiners et al., 2015). The effects of accelerated ageing in COPD lungs has been eloquently reviewed elsewhere (see Barnes et al., 2019; Bateman et al., 2023; Brandsma et al., 2017). Briefly, in the lungs of patients with COPD, there is an accumulation of senescent cells including alveolar epithelial cells, endothelial cells, pulmonary artery smooth muscle cells as well as fibroblasts overexpressing markers of senescence such as p16 (also known as p16INK4a, cyclin‐dependent kinase inhibitor 2A, CDKN2A), p21 (cyclin‐dependent kinase inhibitor 1) and senescence‐associated beta‐galactosidase (SA‐β‐gal) (Amsellem et al., 2011; Noureddine et al., 2011; Tsuji et al., 2006; Woldhuis et al., 2021). This accumulation of lung senescent cells is associated with immunosenescence; a process that attenuates both innate and adaptive immunity, with increased senescence of T‐lymphocytes and macrophages expressing p21 (Hodge et al., 2011; Tomita et al., 2002). It is probable that these markers increase the number of senescent cells leading to small airway fibrosis and alveolar cell loss (i.e., emphysema) in COPD. Subsequently, these senescent cells secrete high levels of pro‐inflammatory cytokines (such as TNF‐α, IL‐1β), growth factors (such as VEGF and TGF‐β) and proteases (such as matrix metalloproteinases MMP9 and MMP12). These SASP mediators further prolong senescence through autocrine and paracrine mechanisms, thereby enhancing senescence within their own cellular environment and/or transmitting senescence to adjacent cells (Barnes et al., 2019; Huang et al., 2022) (Figure 1).

**FIGURE 2:**
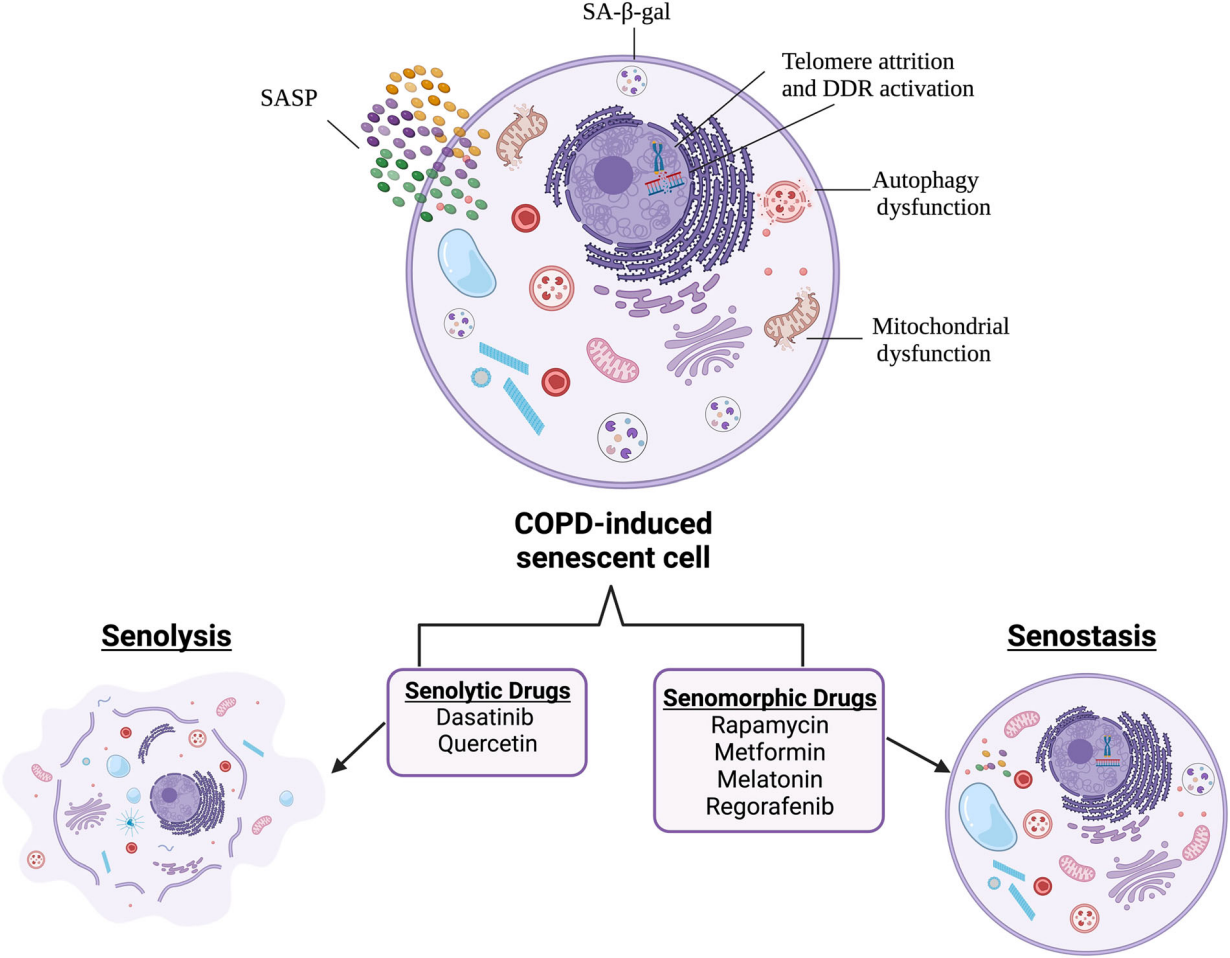
Chronic obstructive pulmonary disease (COPD)‐induced accelerated ageing is associated with a variety of biochemical alterations that drive senescence‐associated characteristics such as enlarged and irregular morphology; an increase in β‐galactosidase activity termed senescence‐associated β‐galactosidase activity (SA‐β‐gal); mitochondrial dysfunction, autophagy dysfunction; telomere attrition and activation of DNA damage response (DDR) pathways; and production of the senescence‐associated phenotype (SASP). There are two types of senotherapeutic agents which can be used to halt or improve COPD and its neuropathological comorbidities. Senolytic drugs selectively remove senescent cells while senomorphic agents attenuate the pro‐inflammatory secretory phenotype (SASP) to induce senostasis.

The question remains, how do lung senescent cells drive neurological and neurodegenerative comorbidities? We propose that the metabolically active senescent cells in the COPD lungs secrete an over‐exuberant SASP profile (via secretion of multiple inflammatory and oxidative stress proteins) into the systemic circulation, via extracellular vesicles, driving senescence in distal organs, resulting in the comorbidities discussed above. Pulmonary extracellular vesicles in individuals with COPD consist of microvesicles that emerge from plasma membranes, as well as exosomes originating from the endoplasmic reticulum, which carry SASP proteins (Lacedonia et al., 2016). These vesicles are emitted by cells and taken up by other neighbouring cells, thus participating in intercellular communication and capable of conveying their cargo to recipient cells either by means of direct interaction or by being internalised by the recipient cells (Lacedonia et al., 2016). Pulmonary extracellular vesicles can enter the systemic circulation in patients with lung disease (Kadota et al., 2018). As such, the over‐exuberant SASP profile from lung senescent cells could be transported to other organs through the circulation leading to the comorbidities of COPD (Reid et al., 2021). In this review, we focus on discussing the evidence supporting COPD‐induced cellular senescence in the CNS and potential therapeutic agents that may halt or improve disease progression (Figure 2).

## 4 COPD‐INDUCED AGEING WITHIN THE CNS

### 4.1 Senescence‐associated secretory phenotype

Senescent cells exhibit an elevated neuroinflammatory profile characterised by the increased expression of pro‐inflammatory cytokines, their regulators, MMPs and oxidative stress mediators, collectively known as SASP (Kumari & Jat, 2021). Within these senescent cells, the SASP response is mediated by p21^CIP1^, which inhibits cyclin/CDK complexes leading to the activation of p38 mitogen‐activated protein kinase and Janus‐activated kinases (JAK). This, in turn, triggers the activation of the transcription factor NFκB (Kumari & Jat, 2021), resulting in the secretion of pro‐inflammatory cytokines (such as TNF‐α, IL‐1β, IL‐6 and IL‐8), chemokines (such as CXCL1, CXCL8 and CCL2) and MMPs (such as MMP2 and MMP9) (Kumari & Jat, 2021). In aged mice, inhibition of JAK pathways suppressed SASP and alleviate frailty (Xu et al., 2015). Thus, SASP components and their inducing factors play an important role in the cellular senescence profile.

However, the question remains, which CNS cell types are undergoing senescence and inducing the SASP profile in people with COPD? Astrocytes are known to play a crucial role in brain physiology and neuronal function by providing support for neuronal cells, maintenance of ion homeostasis via regulating potassium ions, control glutamatergic transmission within the brain and maintain BBB integrity. However, during healthy ageing, astrocytes lose their neuronal support and reduce interactions with synapses due to a reduction in glutamate transporter genes (Ojo et al., 2015). Moreover, in vitro studies have shown that external stressors induced astrocyte cellular senescence, with an increase in p21, p53 and SA‐β‐gal (Bitto et al., 2010; Pertusa et al., 2007). In post‐mortem human brains, the frontal cortex of both older and Alzheimer's disease patients expressed a greater burden of p16‐positive astrocytes, compared with young subjects (Bhat et al., 2012). In addition, with age, astrocyte morphology is altered and the cells become reactive and secrete a number of cytokines and chemokines that overlap with SASP factors (including TNF‐α, IL‐1β, IL‐6 and IL‐8) (Dai et al., 2020). Therefore, it is highly likely that astrocytes are, at least in part, involved in regulating the function of the aged brain and are partly responsible for the senescence phenotype within the brain (Dai et al., 2020). Alongside astrocytes, microglia may also undergo senescence and contribute to the SASP profile within the brain. In the healthy young brain, microglial cells typically display a surveillant ramified morphology. However, in the aged brain, microglial cells undergo morphological changes, appearing to have a more dystrophic morphology displaying a decreased rate of process movement and migration to tissue injury (Wong, 2013). These substantial morphological and functional changes were associated with a sustained exacerbated inflammatory response, in line with SASP cytokine profiles (TNF‐α, IL‐1β, IL‐6) (Wong, 2013).

Oxidative stress, as a result of cigarette smoke exposure, is likely to be a crucial inducer of the SASP response and, ultimately, the cellular senescence profile that develops in the lungs of COPD patients (Barnes et al., 2019), as well as in the CNS. Within the brain, exposure of rodents to cigarette smoke is associated with increased gene expression of pro‐oxidants such as iNos, Nox4, and Nox2 in the whole brain (Anbarasi et al., 2006; Khanna et al., 2013; Sivandzade et al., 2020) and in cognitive‐dependent regions including the hippocampus (De Luca et al., 2022; Dobric et al., 2022; Zhang et al., 2022). Given that excessive oxidative stress is often linked to an over‐exuberant pro‐inflammatory profile, it is tempting to speculate that the over‐exuberant oxidative stress profile in the COPD lungs and brain, is accompanying the accelerated SASP responses. Acute exposure to cigarette smoke in rodents for 3–6 weeks increases the gene expression of *Nfkb*, *Il6*, *Il1b* and *Tnfa* (Khanna et al., 2013; Sivandzade et al., 2020). However, it must be noted that these studies have assessed whole brain tissue, rather than individual regions. More recently, Prasedya et al. showed increased expression of TNFα protein, following 14 days of cigarette smoke exposure in mice, compared to controls (Prasedya et al., 2020). We have recently shown significantly higher levels of both cytokines (*Tnfa*, *Il1b*), *Csf1* chemokine and *Mmp9* in the hippocampus of mice exposed to cigarette smoke, compared to aged‐matched control mice (De Luca et al., 2022). Accordingly, in our model, the increased pro‐inflammatory profile was associated with sustained hippocampal microglial activation (De Luca et al., 2022; Dobric et al., 2022). These microglia remain in an amoeboid morphology following long‐term cessation, suggesting that hippocampal microglia from cigarette smoke‐exposed mice may have undergone stress‐induced senescence (De Luca et al., 2022). This is consistent with previous data showing that an acute treatment of mice, with the tobacco‐specific pro‐carcinogen compound, 4‐N‐methyl‐N‐nitrosamino‐1‐(3‐pyridyl)‐1‐butanone, for as little as 4 days, can induce robust changes in hippocampal microglial morphology (Ghosh et al., 2009). We have also shown that chronic cigarette smoke exposure induces a reactive astrocyte profile within the hippocampus (Dobric et al., 2022) and hypothalamus (De Luca et al., 2023). This reactive astrocyte profile was associated with endothelial cell tight junction protein impairment (reduction in ZO‐1), thus reducing BBB integrity, compared to sham mice, leading to working memory impairments (Dobric et al., 2022). Interestingly, treatment with apocynin, a NAPDH inhibitor, was sufficient to attenuate both lung inflammation and hypothalamic microglial activation, but not reactive astrocyte profiles, following cigarette smoke exposure (De Luca et al., 2023). Thus, the increasing SASP milieu within the brain may be driven by both the systemic and brain‐derived changes of the immune cells because of the over‐exuberant pro‐inflammatory and oxidative stress mediators that may enter via extracellular vesicles from the COPD lung.

### 4.2 Telomere attrition and DNA damage

The dynamic process of cellular senescence is usually driven by the progression from a transient to a stable cell‐cycle arrest which occurs through telomere attrition and the sustained activation of DNA damage response (DDR) pathways (Bhat et al., 2012; Boccardi et al., 2015). Telomere attrition reduces the protection of chromosomal ends, exposing them to DNA damage, inducing the inability to perform DNA repair, thus further propagating DDR. Telomeres are ribonucleoprotein structures located at chromosomal ends that are essential to preserve and maintain genome stability during DNA replication (Astuti et al., 2017; Barragán et al., 2021; Blackburn, 2001; O'Sullivan et al., 2010). Human telomeres inevitably shorten during replication and cells will cease proliferating and become senescent; thus, telomere length has long been considered a marker of cellular ageing (Bernadotte et al., 2016; Fyhrquist et al., 2011). Moreover, shorter telomeres have been associated with mortality and increased rates of age‐related diseases, as well as being linked to increased risk and prognosis of stroke (Tian et al., 2019; Wang et al., 2021). A recent clinical study showed that longer telomeres protected against the risk of Alzheimer's disease and increased life expectancy (Rodríguez‐Fernández et al., 2022). However, other studies have found no changes in telomere length in people living with Alzheimer's disease, compared with healthy aged‐matched controls (Hinterberger et al., 2017). Moreover, DNA damage and the cellular senescence that ensues has been demonstrated to play an important role in the onset and aggravation of Alzheimer's disease (Bhat et al., 2012; Boccardi et al., 2015). Of particular interest, increased senescence has been found in different brain cell types including microglia, astrocytes and neurons, as shown by the enhanced expression of p53 expression, a mediator of cell stress regulation, DNA damage (Myung et al., 2008), telomere attrition or damage and SA‐β‐gal (Flanary & Streit, 2005). Moreover, plasma samples from patients with Alzheimer's disease showed increased levels of senescent cells with higher SA‐β‐gal and p53 levels (Caldeira et al., 2017). While telomere shortening has been proposed as a valuable predictor of Alzheimer's disease incidence, its association with other neurodegenerative diseases such as Parkinson's disease, amyotrophic lateral sclerosis and frontotemporal diseases remains unknown (Rodríguez‐Fernández et al., 2022). Further studies are necessary to elucidate the function of telomere length in neurodegenerative diseases and its efficiency as a biomarker of cellular ageing.

Given that people with COPD have an increased incidence of both neurological disorders and neurodegenerative diseases, it is possible that there is a causal relationship between telomere length, DNA damage and neurological outcomes in this population. Recent clinical studies illustrate that smokers have a greater telomere attrition in their blood leukocytes, compared to non‐smokers, accounting for approximately 0.64%–1.23% telomere variation (Sulastri & Lestari, 2017). This difference in telomere length could be between 50 and 190 base pairs shorter than in non‐smokers (Wulaningsih et al., 2016). Interestingly, a longitudinal study of 5624 American participants observed that smoking status was associated with a reduction in telomere length over a 16‐year follow‐up (Zhang et al., 2016). This association was attenuated in men and was linked with a higher rate of smoking cessation in men than women (Zhang et al., 2016).

Interestingly, markers of oxidative damage to DNA and RNA, such as the oxidised nucleosides, 8‐hydroxydeoxyguanosine (8OHdG) and 8‐hydroxyguanosine (8‐OHG), are increased in neurons in post‐mortem brains of people with a confirmed Alzheimer's disease diagnosis (Nunomura et al., 2001). The concentration of 8‐OHdG in the cerebrospinal fluid of Alzheimer's disease patients was positively correlated with the duration of illness (Isobe et al., 2010). In a preclinical cigarette smoke‐exposure model of COPD, levels of 8‐OHG in the hippocampus of rats exposed to cigarette smoke were increased, compared with those in control rats (Ho et al., 2012). Furthermore, 12 weeks exposure to cigarette smoke in Wistar rats induced breaking of double‐stranded DNA in the cerebral cortex alongside cell death (assessed via comet assay and Fluoro‐Jade C, respectively) (Naha et al., 2017). Although the increased prevalence of telomere attrition and DNA damage has not specifically been assessed in COPD cohorts, but in human smokers and/or preclinical models, it is likely that the cellular and oxidative stress induced by cigarette smoking, leads to progressive shortening of the telomeres and the activation of DDR pathways resulting in replicative senescence (i.e., normal cell cycle arrest) and an increased risk of stroke and Alzheimer's disease, but possibly no other neurogenerative diseases.

### 4.3 Autophagy in the COPD brain

Senescent cells exhibit a complex phenotype, with growing evidence for a connection between defective autophagy, lysosome function and the senescence process. Autophagy plays a crucial role in cellular homeostasis, enabling cells to remove damaged or unnecessary organelles and molecules. Recent reviews have focused on the role of autophagy in CNS ageing (Sikora et al., 2021). Autophagic activity (and accumulation of autophagic vesicles) within the brain decrease with age, in both experimental models and post‐mortem human brains, even in the absence of disease (Sikora et al., 2021). Autophagic function is dependent on lysosomes and, thus, dysfunctional lysosomes can lead to impaired autophagy and the leakage of lysosomal contents into the cytosol, a phenomenon known as lysosomal membrane permeabilization. During oxidative and senescence conditions, neurons accumulate lipofuscin aggregates; intralysosomal pigments formed by lipids, metals and misfolded proteins; which can decrease autophagy. Additionally, the accumulation of autophagic vesicles has been associated with decreased fusion of autophagosome with lysosomes (Yoshii & Mizushima, 2017). Neural progenitor cells have elevated markers of autophagic vesicles, marking impaired autophagic flux (Sunderland et al., 2020). Moreover, activation of autophagy has been linked to increased SASP manifestations. SASP cells release high levels of inflammatory cytokines, immune modulators, growth factors, and proteases. In non‐senescent cells, the transcription factor GATA4 is normally degraded by autophagy, and inhibition of this degradation results in the activation of a key SASP regulator, NF‐κB (Kang et al., 2015). Collectively, it is likely that autophagy dysfunction induced by lysosome impairment is closely connected with senescence in the CNS.

Despite these advances, the published evidence for the association between cigarette smoking, COPD phenotype, senescence, and autophagy in brain cells remains limited. There are major alterations in lipid metabolic pathways in people with COPD, with an increase in lipids within the alveolar macrophages (Chen et al., 2019; Fujii et al., 2021). Exposure to cigarette smoke increased in hippocampal autophagic markers, such as Beclin‐1, ATG7, LC3‐I and LC3‐II, alongside apoptotic markers (Huang et al., 2013). While we have not specifically evaluated the autophagy profile within the brain, our research has shown that both acute (8 weeks) and chronic (24 weeks) exposure to cigarette smoke is sufficient to induce hippocampal and hypothalamic microglial activation, along with an increase in lipid oxidative stress (De Luca et al., 2022, 2023; Dobric et al., 2022). Thus, it is possible that such heightened lipid oxidative stress may contribute to an impairment of lipid metabolism and serve as an indicator of autophagy impairment. However, further investigation is necessary to fully understand these mechanisms.

The senescent signature originating from COPD lungs has the potential to initiate a disruption in BBB function and integrity, thereby triggering an exaggerated inflammatory milieu within the CNS and, as a consequence, amplifying the senescent milieu within this environment. The peripheral inflammatory and oxidative environment activates astrocytes and microglia to undergo cellular senescence, releasing SASP factors that contribute to telomere attrition and DNA damage. The intricate interplay between cigarette smoking, COPD and pulmonary senescence, along with their implications for the CNS, remains an under‐explored area of research, warranting comprehensive investigation to unveil the complexities of these mechanisms. Nonetheless, discerning and targeting the specific components of COPD‐induced pulmonary SASP that evoke senescence within the brain holds the potential to unlock novel therapeutic avenues aimed at mitigating COPD‐associated neurological and neurodegenerative comorbidities.

## 5 IS CIRCADIAN RHYTHM DISRUPTION THE MISSING LINK IN COPD‐INDUCED ACCELERATED AGEING?

There is accumulating evidence that key pathological processes and phenotypes in both the clinical and preclinical COPD settings are related to daily pulmonary circadian rhythm changes leading to a senescence profile within the lungs (see Giri et al., 2022). The circadian clock, which is an endogenous time‐keeping system, allows for synchronisation of physiological and behavioural processes over ~24 h by core circadian clock genes both within the CNS (the suprachiasmatic nucleus of the hypothalamus) and peripheral organs (Ahmed et al., 2022). The primary transcriptional–translational feedback loop consists of a circadian locomotor output cycle kaput (CLOCK) and Brain and Muscle ARNT‐like protein‐1 (BMAL1) heterodimer complex (Ahmed et al., 2022). The CLOCK:BMAL1 complex translocates to the nucleus activating the expression of Period (PER1/2/3) and Cryptochrome (CRY1/2) proteins, forming a heterodimer complex and repressing their own transcription by acting on the CLOCK:BMAL1 complex (Ahmed et al., 2022). Circadian clock disruption with a period and a phase shift have been implicated during both replicative and oxidative stress‐induced premature senescence in primary human lung fetal fibroblast (TIG‐3) cells, with increased p21 and SA‐β‐gal gene expression (Ahmed et al., 2019, 2021). Moreover, senescent cells are metabolically active and many of the molecular pathways and factors disrupted in these cells are known to affect the cellular clock. For example, the cell cycle arrest marker; p53, regulates the binding of *Per2* to the E‐box element in the PER2 promoter and pharmacological stabilisation of p53 stabilises the *Per2* gene expression while p53^−/−^ mice have reduced locomotor activity periods indicative of circadian rhythm disruption (Ahmed et al., 2022). In addition, upregulated expression of mTOR (mammalian target of rapamycin) is associated with autophagy and inhibition of mTOR induces lengthened circadian periods and alters animal behaviours (Ahmed et al., 2022).

Dysfunction of the circadian rhythm clock promoted progression of COPD within the lung, via inflammatory and oxidative stress responses, leading to worsened symptoms and severity of the disease in the early morning or late night including reduced lung function (Scichilone et al., 2019; Tsai et al., 2019), nocturnal oxygen desaturation (McNicholas et al., 2013) and cardiac arrhythmias (McNicholas et al., 2013). Rahman and colleagues have shown lung function and inflammatory profiles are altered during both day and night in mice exposed to cigarette smoke. This is associated with decreased expression of core clock genes (*Bmal1*, *Nr1d1* [coding for Rev‐erbα] and *Per1*) in the lungs, along with enhanced lung inflammation and altered lung function (Hwang et al., 2014; Wang et al., 2021). Alongside this, Rev‐erbα regulates lung fibrotic progression via stabilising collagen and TGF‐β‐induced lysyl oxidase expression in human lung fibroblasts (Wang et al., 2023). Gebel et al. have shown that the expression of core circadian rhythm genes is changed in rodents exposed to both cigarette smoke and pro‐inflammatory mediators (lipopolysaccharide or TNF‐α); however, this may be due to a shift in the timing and amplitude of these genes (Gebel et al., 2006). In the clinical setting, the number of Bmal1‐positive cells was decreased in the lungs of individuals with COPD which may partly result from the regulation of acetylation and degradation of BMAL1 and PER2. Within the lungs, the altered circadian clock molecules (Bmal1 and Per2) are linked to impaired autophagy, with lower activity of autophagy‐related proteins including mTOR and Sirtuin‐1 (SIRT1) in lung epithelial cells and macrophages of humans and mice (Sundar et al., 2015; Tran et al., 2015). Moreover, patients with COPD exhibited lower levels of BMAL1 and CLOCK in plasma (Li et al., 2022). Li and colleagues demonstrated that exposure to cigarette smoke extract increased cell senescence and decreased CLOCK and BMAL1 expression in human bronchial epithelial cells, while overexpression of BMAL1 and CLOCK inhibited senescence through the MAPK pathways, suggesting that BMAL1 or CLOCK deficiency may contribute to the development of COPD by promoting cellular senescence in the lung (Li et al., 2022).

Collectively, these results suggest that the pulmonary circadian clock becomes less robust and flexible in people with COPD. However, whether this is associated with accelerated cellular senescence and circadian rhythm disruption within the brain remains unknown. To the best of our knowledge, we were the first to demonstrate that, in mice, chronic exposure to cigarette smoke leads to a notable shift in circadian rhythm regulation profiles (reduced locomotor activity, respiratory exchange ratio [RER] and energy expenditure during the dark phase but increased VO_2_), increased expression of the transcriptional repressor gene, *Per2*, and a reduced number of BMAL1‐positive cells, compared with data from sham mice (De Luca et al., 2023). Within our preclinical cigarette smoke exposure model, we have previously shown a SASP milieu within the hippocampus and hypothalamus, with increases in pro‐inflammatory cytokines and chemokines, oxidative stress markers as well as increased microglial and astrocyte activation (De Luca et al., 2022, 2023; Dobric et al., 2022). Thus, we show a possible link between the circadian clock and cellular senescence (i.e., SASP profiles), which may promote COPD‐induced accelerated ageing (Figure 1). Understanding the interrelationship between circadian clock and cellular senescence in COPD is desperately needed to help advance the development of novel clock‐based therapeutic agents.

## 6 POSSIBLE SENOTHERAPEUTIC TREATMENTS FOR COPD‐INDUCED ACCELERATED AGEING

### 6.1 Senomorphic treatments

Currently, there are no effective therapies to attenuate COPD disease progression, however, there is a variety of therapeutic agents that alleviate symptoms and enhance quantity of life. These therapies include smoking cessation, pulmonary rehabilitation, bronchodilator therapy and oxygen supplementation (Barnes et al., 2019). Senomorphic therapies regulating the molecular pathways controlling senescence may lead to new therapeutic strategies to improve both lung and extra‐pulmonary comorbidities (Table 1 and Figure 2).

**TABLE 1:** Effect of senotherapies in chronic obstructive pulmonary disease.

Drug	Proposed mechanism(s)	Effects in the COPD lung	Reference
Rapamycin rapalogs	mTOR complex inhibitor	↓ mTOR activity and corticosteroid sensitivity in PBMC. ↓ mTOR activity and lung cell senescence in mice and ↓senescence and SASP in human COPD lungs.	(Houssaini et al., 2018; Mitani et al., 2016)
Metformin	AMPK activation	↓ senescence and SASP profiles and ↓ airway space in mouse model of emphysema. In diabetic COPD patients, ↓ blood CRP and glucose levels during AECOPD. Unable to ↓ blood CRP and glucose levels during AECOPD in non‐diabetic COPD patients.	(Garnett et al., 2013; Hitchings et al., 2016)
Melatonin	Antioxidant and mTOR complex inhibitor	↓ pulmonary oxidative stress, dyspnoea, and IL‐8 levels, ↑ sleep duration and efficacy. ↓ airway inflammation by inhibiting the NLRP3 inflammasome and IL‐1β in lungs in COPD mouse model.	(de Matos Cavalcante et al., 2012; Halvani et al., 2012; Nopparat et al., 2010)
Quercetin	Senolytic and antioxidant	↓ lung dysfunction, ↓ SASP (leukocyte, oxidative stress) in mice. ↓ alveolar chord length, ↓ SASP in mice. ↓ rhinovirus‐induced lung inflammation. Safely tolerated in mild‐to‐severe COPD.	(da Silva Araújo et al., 2020; Ganesan et al., 2010; Han, Barreto, et al., 2020)
Quercetin and dasatinib	Senolytics	No COPD studies reported. ↓ pulmonary senescence markers and SASP and improved the physiological functions in idiopathic pulmonary fibrosis (IPF). ↑ α‐Klotho protein in urinary samples from IPF patients.	(Hickson et al., 2019; Justice et al., 2019; Zhu et al., 2022)

Abbreviations: AECOPD, acute exacerbation of chronic obstructive pulmonary disease; COPD, chronic obstructive pulmonary disease; mTOR, mammalian target of rapamycin; SASP, senescence‐associated secretory phenotype.

Despite our understanding that excessive inflammation and oxidative stress induces dysregulated expression and oscillation of circadian clock genes in COPD lungs, it remains unknown whether targeting the circadian rhythm may reverse and/or prevent accelerated ageing of the respiratory system and neuropathological comorbidities associated with COPD. The negative regulator of autophagy, mTOR, is involved in the inflammatory responses in the COPD lungs. Thus, upon upregulation of mTOR expression, inflammatory markers are activated resulting in airway remodelling and damage to the lung parenchyma (Hu et al., 2021). The core clock protein, Per2, can inhibit mTOR activity, and thus inhibit autophagy. Therefore, therapeutic agents targeting the circadian rhythm could reverse and/or prevent premature ageing, SASP profile, and autophagy dysregulation (Giri et al., 2022; Hu et al., 2021).

#### 6.1.1 Rapamycin

Rapamycin is currently the most effective pharmacological approach to directly target the ageing process increasing life span in the preclinical setting. Rapamycin binds to FKBP12 (FK506 binding protein 12) and inhibits the activity of the mTOR complex (Johnson et al., 2015). Rapamycin is an FDA approved drug for organ transplantations and is currently undergoing clinical trials assessing the safety and efficacy in reducing clinical measures of ageing in the elderly population (Hahn et al., 2019). Interestingly, organ transplantations are associated with an increased risk of non‐melanoma skin cancers. However, numerous clinical studies have demonstrated that rapamycin reduced the incidence of cancers in patients following organ transplantation (Karia et al., 2016), even when given in combination with cyclosporine (Mathew et al., 2004). However, there is currently no clinical trial for rapamycin or other mTOR inhibitors in individuals with COPD or neurodegenerative diseases such as Alzheimer's disease. Rapamycin attenuated mTOR activity in ex vivo peripheral blood mononuclear cells from individuals with COPD and in vitro studies, using U937 cells, effectively inhibit mTOR activity and c‐JUN expression, and restored corticosterone sensitivity following cigarette smoke exposure (Mitani et al., 2016). Houssaini and colleagues developed transgenic mice models of mTOR overactivation in (i) lung vascular cells and (ii) alveolar epithelial cells inducing rapid development of lung emphysema and pulmonary hypertension, supporting the causal relationship between COPD, lung cell senescence and mTOR activation (Houssaini et al., 2018). They also showed that rapamycin effectively inhibits senescence and SASP (reduced IL‐6, IL‐8 and CCL2) in cells derived from people with COPD (Houssaini et al., 2018); however, circadian rhythm markers were not assessed. Although Saito et al. did not assess the inhibition of mTOR by rapamycin, they demonstrated that cigarette smoke‐induced Lamin B reduction and downstream inhibition of the mTOR regulator, DEPTOR in murine lung airway epithelial cells and COPD lungs resulting in mTOR activation, mitochondria accumulation and cellular senescence (Saito et al., 2019). Therefore, further suggesting that rapamycin may influence mTOR signalling and improve senescence in COPD pathogenesis. Alongside this, numerous studies have shown the beneficial effects of rapamycin in animal models of neurodegeneration and ageing, particularly Alzheimer's disease. Using various mouse models of Alzheimer's disease, we now know some beneficial outcomes of rapamycin treatment including, but not limited to, reduction of both amyloid‐β deposition and tau phosphorylation, restoration of cerebral blood flow and cerebromicrovascular density, preservation of the BBB integrity, and restored cognitive function (Kolosova et al., 2013; Lin et al., 2017; Siman et al., 2015; Van Skike et al., 2018). Thus, inhibition of mTOR via rapamycin may be a potential novel therapeutic target for COPD and its neurological comorbidities. However, it should be noted that despite the beneficial effects of rapamycin for both COPD and neurodegenerative diseases, rapamycin commonly induces adverse effects such as mouth ulceration, pneumonitis and delay in wound healing.

#### 6.1.2 Metformin

Metformin is a biguanide, anti‐glycemic drug that is commonly used to treat Type 2 diabetes mellitus. It is characterised as a ‘geroprotector’ as it has been shown to reduce mortality and ageing‐related diseases, independent of diabetes regulation (Campbell et al., 2017). This drug activates AMPK (5′ AMP‐activated protein kinase) which inhibits the Sirtuin‐mTOR signalling pathway (Campbell et al., 2017). AMPK modulates the phosphorylation of PER2 and CRY1; thus, a single dose of metformin shortens peripheral circadian clocks, while chronic treatment in ob/ob mice does not disrupt either the liver or adipose tissue circadian clocks (Hasan et al., 2022). Using an elastase‐induced emphysema mouse model, Cheng and colleagues showed that metformin was able to reduce SASP molecules, IL‐6 and IL‐8, alongside reduced enlarged airspaces (Cheng et al., 2017). Metformin limits hyperglycaemia‐induced bacterial growth; thus, it is thought that treatment with metformin may prevent and treat respiratory infections in people with COPD (Garnett et al., 2013). However, Hitchings et al observed that in non‐diabetic COPD patients, short‐term metformin was unable to reduce the CRP and blood glucose levels during a severe exacerbation of COPD induced by either bacterial and/or viral infection (Hitchings et al., 2016). On the other hand, people with comorbid COPD and diabetes treated with metformin had fewer exacerbations of COPD, were less sick, less likely to require oxygen and had reduced mortality (Bishwakarma et al., 2018; Ho et al., 2019). Despite the beneficial health outcomes in diabetic COPD patients, metformin has been associated with lactic acidosis, raising doubts about its safety and efficacy in non‐diabetic, COPD patients.

#### 6.1.3 Melatonin

Melatonin is an endogenous hormone produced in the pineal gland and is thought to have antioxidant properties (de Matos Cavalcante et al., 2012; Halvani et al., 2012). Melatonin is known to support the maintenance of the circadian clock and regulate autophagy precursors. In patients with COPD, endogenous melatonin is decreased during acute exacerbations alongside an imbalance of oxidative stress and antioxidant enzymes (de Matos Cavalcante et al., 2012). Melatonin supplementation in a randomised, double‐blinded controlled trial showed a reduction in pulmonary oxidative stress, dyspnoea and IL‐8 levels as well as improvement in sleep duration and efficacy (de Matos Cavalcante et al., 2012; Halvani et al., 2012). In preclinical models of COPD, supplemental melatonin alleviated airway inflammation by specifically inhibiting the NLRP3 inflammasome and IL‐1β (Nopparat et al., 2010). The antioxidant properties of melatonin are also known to inhibit mTOR activation and the JNK pathway to reduce autophagy via regulation of the circadian clock genes (Kijak & Pyza, 2017; Yoshida et al., 2010). Thus, improvements in both pulmonary function and sleep regulation (i.e., improved circadian rhythm) could be an indication of reduced cellular senescence profiles, thereby identifying a potentially beneficial therapeutic avenue for COPD.

#### 6.1.4 Regorafenib

Regorafenib is an FDA‐approved inhibitor that targets several receptor tyrosine kinases. It exhibits distinct biological activities that does not induce significant cell death at low concentrations and may therefore be a promising candidate for targeting cellular senescence. Regorafenib has been approved for the treatment of metastatic colorectal and hepatocellular carcinoma and gastrointestinal stromal tumours (Ettrich & Seufferlein, 2018; Kehagias et al., 2022; Strumberg et al., 2012). In a study conducted by Han and colleagues, using a 5X FAD mouse model of Alzheimer's disease, regorafenib demonstrated its potential by increasing dendritic spine density and reducing levels of amyloid‐β plaques. This effect was achieved through modulating the processing of amyloid precursor protein and associated proteins, thus improving the pathology of Alzheimer's disease (Han, Kang, et al., 2020). Moreover, a recent study by Park et al. identified the effectiveness of a sublethal dose of regorafenib in attenuating cellular senescence in human lung fibroblast cells (IMR‐90 cells). Treatment with regorafenib successfully mitigated the βPIX knockdown‐ and doxorubicin‐induced senescence and replicative senescence and reduced SA‐β‐Gal staining and the SASP profile (Park et al., 2023). In complementary studies, the potential of regorafenib was shown in a porcine pancreatic elastase (PPE)‐induced model of emphysema. Treatment with regorafenib (5 mg·kg^−1^) attenuated the senescence profile with reduced SA‐β‐Gal staining and p16^INK4a^ expression and improved PPE‐induced emphysema in mice (Oh et al., 2023; Park et al., 2023). To the best of our knowledge, there is no experimental data on the effects of regorafenib on the circadian rhythm in a disease setting. Despite this, these findings provide new insights for regorafenib as a novel senomorphic drug with potential therapeutic applications in COPD and its associated neurological comorbidities.

### 6.2 Senolytic treatments

Senolytic drugs can selectively remove senescent cells (alongside decreased SASP profiles and increased efficacy of stem and progenitor cells for tissue repair) while having minimal effect on proliferating cells. Thus, senolytic drugs are a promising therapeutic avenue to treat COPD and its associated neuropathological comorbidities (Table 1 and Figure 2). Over the last decade, several transgenic mouse models have been developed eliminating senescent cells in vivo, delaying age‐related functional impairments and extending life span (Baker et al., 2011). In INK‐ATTAC mice, activation of the inducible caspase‐like transgene via the p16^INK4A^ promoter induced apoptosis of senescent cells and delayed age‐related pathologies including cataracts, sarcopenia, and loss of adipose tissue (Zhu et al., 2016). Using siRNA to silence apoptotic pathways, Zhu et al. demonstrated that senescent cells display increased expression of pro‐survival networks leading to apoptotic resistance (Baker et al., 2020). Thus, senolytic treatments targeting these pathways may be a promising therapeutic avenue. Current potential senolytic treatments include but are not limited to, BCL‐2 inhibitors, heat shock protein 90 (HSP90), dasatinib and quercetin.

Senescent cells rely on their anti‐apoptotic pathways to protect them from apoptosis including BCL‐2/BCL‐XL family members, HIF‐1α and P13K/Akt pathways (Zhu et al., 2015). Thus, BCL‐2 is upregulated in the lungs of COPD patients which is associated with the over‐exuberant inflammatory profile (Siganaki et al., 2010) and promotes autophagy in human bronchial epithelial and human alveolar epithelial cells (Qin et al., 2019). Moreover, cigarette smoke exposure led to an increase in hippocampal apoptotic markers in a rodent model (Huang et al., 2013). During senescence, the BCL‐2 family is upregulated while inhibition or down‐regulation in senescent cells induces apoptotic cell death. Navitoclax, an orally bioavailable, pan‐BCL inhibitor, selectively induced apoptotic cell death of lung fibroblast cells (IMR‐90 cells) in vitro (Zhu et al., 2016). Moreover, navitoclax reduced the senescent phenotype in aged haematopoietic stem cells in mice (Chang et al., 2016). More recently, the efficacy and safety of navitoclax have been assessed in a Phase II trial in combination with ruxolitinib therapy for the treatment of myelofibrosis (Harrison et al., 2022). This combination resulted in reduced spleen volume and improved bone marrow fibrosis but the trial did not investigate cellular senescent parameters.

Dasatinib and quercetin decreased senescent cells in aged mice and alleviated symptoms in age‐related diseases including cardiac function and carotid vascular reactivity (Kirkland & Tchkonia, 2020; Roos et al., 2016). Moreover, the senolytic cocktail of dasatinib and quercetin improved the physiological functions in patients with IPF and diabetic kidney disease. This beneficial effect was associated with the elimination of p16^INK4A^‐positive cells, attenuated SASP proteins in the blood and reduced activity of SA‐β‐gal (Hickson et al., 2019; Justice et al., 2019). α‐Klotho was also decreased in senescent cells and a recent study has shown that removing senescent cells via dasatinib and quercetin treatment increased α‐klotho protein in urinary samples from IPF patients, thus, suggesting that urinary α‐klotho assessments may be useful to assess senolytic activity in clinical trials (Zhu et al., 2022). More recently, a Phase I, single‐blind, randomised trial demonstrated that intermittently dosing IPF patients with dasatinib and quercetin is feasible and generally well‐tolerated (Nambiar et al., 2023). To the best of our knowledge, this senolytic cocktail has not been investigated in either preclinical models or in humans with COPD, however, treatment with quercetin alone has recently been studied. Quercetin is a flavonoid compound shown to modulate inflammation and redox imbalance. Prophylactic treatment with quercetin prevented the increased leukocytes, oxidative stress and lung dysfunction in mice exposed to cigarette smoke for five days (da Silva Araújo et al., 2020). Quercetin also prevented the progression of emphysema with improved elastic recoil and decreased alveolar chord length along with decreased oxidative stress, lung inflammation and expression of MMP9 and MMP12 in elastase/LPS‐exposed mice, compared to vehicle treated controls (Ganesan et al., 2010). A randomised, double‐blinded controlled trial showed that quercetin was safely tolerated in COPD patients with mild‐to‐severe lung disease with no drug‐related severe adverse outcome (Han, Barreto, et al., 2020). While further studies are required to assess whether this drug can improve pulmonary cellular senescence, it does provide evidence for a safely tolerated novel senolytic therapy.

The study of senotherapeutic drugs presents a fascinating avenue in the pursuit of combatting ageing and disease‐related issues. Senolytic compounds exhibit a unique ability to selectively trigger apoptosis in senescent cells, while sparing non‐senescent cells (Hickson et al., 2019; Justice et al., 2019), whereas senomorphic compounds suppress markers of senescence. However, an essential caveat arises from their lack of discrimination between naturally ageing cells and those driven into senescence by disease (Hickson et al., 2019; Justice et al., 2019). To increase the efficacy and safety of senotherapeutic interventions, further research should focus on the identification of highly specific and sensitive biomarkers that distinguish between cells undergoing senescence as a result of diseases and those in the natural course of ageing. By identifying the unique senescent pathways and characterising the subset of cells involved in these processes, we can develop targeted senolytic treatments to enhance both pulmonary and neuropathological outcomes in patients with COPD.

## 7 CONCLUSION

COPD induces both pulmonary and systemic damage and chronic inflammatory states, which we believe could be a major contributing factor to cellular senescent profiles within the brain leading to neurological and neurodegenerative diseases. Potential therapeutic strategies to benefit those affected could include senomorphic and senolytic therapies to target both the lung and systemic inflammation and oxidative stress. It is important to note that senolytic therapies, such as dasatinib and quercetin, have been proposed for intermittent treatments only. These therapies decrease the senescent reservoirs and lessen the adverse effects that may occur with continued treatment. However, senomorphic pharmacological therapies such as mTOR inhibitors, such as rapamycin, metformin and melatonin, attenuate the SASP profile and autophagy and improve circadian rhythm and therefore may be used as a continuous therapy, allowing for physiological, but not pathological, cellular senescence. The hypothesised combination therapy of using senolytic agents as an induction treatment and senomorphic agents as the maintenance treatment, proposed by Kellogg and colleagues (Kellogg et al., 2021) may be a novel and beneficial approach to improve the neuropathological comorbidities induced by COPD.

### 7.1 Nomenclature of targets and ligands

Key protein targets and ligands in this article are hyperlinked to corresponding entries in the IUPHAR/BPS Guide to PHARMACOLOGY http://www.guidetopharmacology.org and are permanently archived in the Concise Guide to PHARMACOLOGY 2021 (Alexander, Fabbro et al., 2021a, b, Alexander, Kelly et al., 2021a, b).

## AUTHOR CONTRIBUTIONS

**Simone N. De Luca:** Conceptualization (equal), writing—original draft (lead), writing—review and editing (equal). **Ross Vlahos:** Conceptualization (equal), project administration (lead), resources (lead), writing—original draft (equal), writing—review and editing (equal).

## CONFLICT OF INTEREST STATEMENT

The authors declare no conflict of interest in this study.
